# Nanoscale Double‐Heterojunctional Electrocatalyst for Hydrogen Evolution

**DOI:** 10.1002/advs.202201339

**Published:** 2022-04-24

**Authors:** Yangyang Feng, Yongxin Guan, Enbo Zhou, Xiang Zhang, Yaobing Wang

**Affiliations:** ^1^ CAS Key Laboratory of Design and Assembly of Functional Nanostructures and Fujian Provincial Key Laboratory of Nanomaterials State Key Laboratory of Structural Chemistry Fujian Institute of Research on the Structure of Matter Chinese Academy of Sciences Fuzhou Fujian 350002 P. R. China; ^2^ Chongqing Industry Polytechnic College Chongqing 401120 P. R. China; ^3^ Fujian Science and Technology Innovation Laboratory for Optoelectronic Information of China Fuzhou Fujian 350108 P. R. China; ^4^ University of Chinese Academy of Sciences Beijing 100049 P. R. China

**Keywords:** active sites, double‐heterojunction, electrocatalysts, electron transfer, hydrogen evolution

## Abstract

The active sites and charge/mass transfer properties in electrocatalysts play vital roles in kinetics and thermodynamics of electrocatalysis, and impose direct impacts on electrocatalytic performance, which cannot be achieved by a simplex structure. As a prototype, the authors propose a double‐heterojunctional nanostructure of NiS_2_/Ni_3_C@C containing NiS_2_/Ni_3_C and Ni_3_C/C heterojunctions as a general model to optimize the above issues and boost electrocatalytic performance. During the thermal reorganization, the in situ reaction between NiS_2_ nanoparticles and carbon induces the formation of Ni_3_C between them and constructs tightly contacted two kinds of interfaces among the three components. The TEM and XPS reveal the intimately contacted three components and the as‐constructed interacted dual interfaces, further confirming the formation of a porous double‐heterojunctional nanostructure. Theoretical calculations uncover that the electron density redistribution occurs at Ni_3_C/C interface by spontaneous electron transfer from defected carbon to Ni_3_C and lower ΔG_H*_ achieves at NiS_2_/Ni_3_C interface by the concentrated interfacial charge density, which favors the simultaneous realization of high catalytic activity and rapid charge/mass transfer. When applied for hydrogen evolution reaction (HER), the porous double‐heterojunctional NiS_2_/Ni_3_C@C exhibits excellent HER activity and durability among all pH values. Profoundly, this special double‐heterojunctional structure can provide a new model for high‐performance electrocatalysts and beyond.

## Graphical abstract

Nanoscale double‐heterojunctional NiS_2_/Ni_3_C@C is first proposed for electrocatalysis as a general model to simultaneously achieve high catalytic activity and rapid charge/mass transfer. The DFT calculations uncover that the Ni_3_C/C heterojunction rearranges the electron density and promotes spontaneous electron/mass flow accessible to catalytic active sites, and the NiS_2_/Ni_3_C heterojunction tunes the electron cloud density and enhances the catalytic activity.

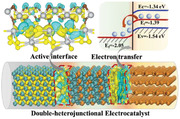

## 1 Introduction

Facing the ever‐growing environmental pollution and an increasingly serious energy shortage, it is urgent to explore environmentally friendly and renewable technologies for environmental remediation and green energy production. Owing to the clean sources, low energy consumption, and valuable products, electrocatalysis emerges as one of the potential technologies.^[^ ^1^ ^]^ As a pivotal component in electrocatalysis, electrocatalysts have a direct impact on electrocatalytic performance.^[^ ^2^ ^]^ Therefore, substantial efforts have been devoted to optimizing the structure of electrocatalysts to enhance the catalytic performance.^[^ ^3^ ^]^ Advanced multistructural electrocatalysts, especially with elaborate design, play vital roles in the kinetics and thermodynamics of electrocatalysis by their special charge/mass transfer properties and sufficient active sites.^[^ ^3^, ^4^ ^]^ Among the various multistructures, heterostructure, where different components meet at the interface, is well‐accepted to adjust the electronic structure by inducing spontaneous electron transfer at the interface and creating more active sites by enlarged exposed edges, mostly exhibiting synergistic performance.^[^ ^5^ ^]^ Recently, various concepts towards heterostructure have been proposed, such as metal–metal, metal–semiconductor, semiconductor–semiconductor, and semiconductor–carbon heterojunctions to enhance the physical, chemical, and electronic properties of catalysts.^[^ ^6^ ^]^ Although the superiorities of the heterojunction, one kind of heterojunction is also limited as the catalytic performance is determined by multiple factors.

Since the double heterostructure laser was first proposed in 1963 by Alferov and Kazarinov,^[^ ^7^ ^]^ double heterostructure ushered in rapid development in various systems among electronics, optics, and light, especially semiconductor crystals and devices, to modify their fundamental parameters, including refractive indices, bandgaps, and electron energy spectrum.^[^ ^8^ ^]^ Currently, double heterostructure where two wider bandgap semiconductor sandwich a smaller bandgap material, are widely applied in optoelectronics due to the low threshold voltage and efficient carrier injection, yet unreported in electrocatalysis.^[^ ^9^ ^]^ For electrocatalysts, heterostructure is an effective strategy to provide sufficient active sites, improve the electrical conductivity and enhance structural stability, synergistically promoting electrocatalytic performance.^[^ ^10^ ^]^ Notably, the electrocatalytic activity of the heterostructure can be greatly enhanced due to the changes of active sites from the components to the interface and the interface electron transfer, which is confirmed and reported by a great number of researches.^[^ ^11^ ^]^ Except for the catalytic activity, the strong interaction at the two‐phase interface can establish electronic communication and generate electronic density redistribution between intimately contacted two components, changing the electronic structure and enhancing the electron reservoir ability.^[^ ^12^ ^]^ The strong electron‐donating component, affected by chemical or physical interactions at the interfaces, acts as an electron reservoir to store and transfer electrons, which plays a vital role in electron density and thus boosts electrocatalytic performance.^[^ ^13^ ^]^ Encouraged by the catalytic activity and electron reservoir ability originating from heterostructure, the synergistic effect of both can absolutely achieve high‐electrocatalytic performance, yet remains unexplored.

Herein, we report a novel nanoscale double‐heterojunctional NiS_2_/Ni_3_C@C catalyst (NiS_2_/Ni_3_C@C) via in situ self‐assembly strategy as a proof‐of‐concept to develop high‐performance electrocatalysts. Different from the traditional multi‐component composites, the in situ formed Ni_3_C coated on the surface of NiS_2_ nanoparticles and encapsulated in line along the porous carbon nanofibers, constructing two kinds of interfaces among NiS_2_, Ni_3_C, and carbon. X‐ray photoelectron spectroscopy (XPS) and high‐resolution transmission electron microscopy (HRTEM) distinctly uncover the two intimately contacted interfaces with strong interaction, confirming the formation of NiS_2_/Ni_3_C and Ni_3_C/C heterojunctions. The charge transfer difference and density of states (DOS) reveal that Ni_3_C/C heterojunction can rearrange electron density and induce spontaneous electron transfer from defected carbon to Ni_3_C and access to active sites, beneficial for fast charge/mass transfer. The charge transfer difference and Gibbs free energy of hydrogen adsorption (Δ*G*_H*_) illustrate that NiS_2_/Ni_3_C heterojunction can tune the electron cloud density and lower the ΔG_H*_, favorable for high catalytic activity. When applied in HER, the porous nanoscale double‐heterojunctional NiS_2_/Ni_3_C@C catalyst yields low overpotentials in a wide pH range with low Tafel slopes and excellent durability.

## 2 Results and Discussion

**Figure 1:**
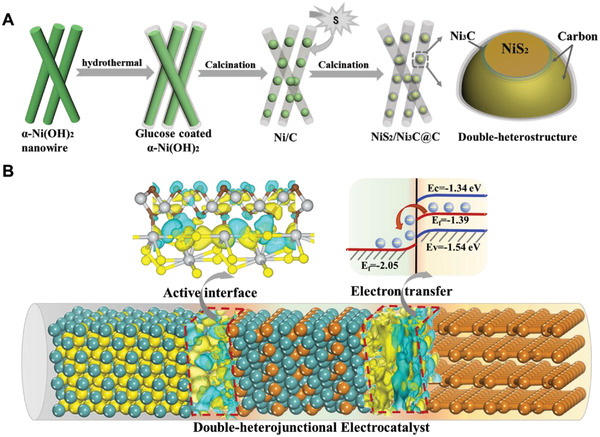
Schematic synthetic route and double‐heterojunctional structure of NiS_2_/Ni_3_C@C. A) Schematic illustration of the formation process. B) The diagram of the two heterojunctions in NiS_2_/Ni_3_C@C.

In a typical synthesis, the novel porous peapod‐like double‐heterojunctional catalyst with NiS_2_/Ni_3_C nanoparticles encapsulated in carbon fibers is fabricated via in situ template strategy from *α*‐Ni(OH)_2_ nanowires^[^ ^14^ ^]^ (Figure S1, Supporting Information) to carbon‐coated Ni nanoparticles (Ni@C) (Figure S2, Supporting Information) and carbon‐coated NiS_2_/Ni_3_C nanoparticles (NiS_2_/Ni_3_C@C) under two‐step calcinations, which finally constructs double‐heterojunctional structure (**Figure** **1**A). In this strategy, we used *α*‐Ni(OH)_2_ nanowires as sacrificial templates to form NiS_2_/Ni_3_C nanoparticles, glucose as a green carbon source to coat on the surface of *α*‐Ni(OH)_2_ with the aid of hydrogen bonding and to transfer into amorphous carbon fibers, finally to construct double‐heterojunctional structure. Ni_3_C layers can be formed on the surface of NiS_2_ nanoparticles due to the strong interaction and in situ chemical reactions at the interface between NiS_2_ and carbon under the calcinations to form NiS_2_/Ni_3_C and Ni_3_C/C heterojunctions. Owing to the high‐temperature calcinations, the outside carbon fiber in NiS_2_/Ni_3_C@C is amorphous (Figure S3, Supporting Information) and porous (Figure S4, Supporting Information). The mesoporous carbon is accepted to exhibit semimetallic properties, which can not only facilitate to form Schottky barrier at the interface of Ni_3_C and C, but also provide numerous ion transfer channels, beneficial for electron/ion fast exchange. Figure 1B shows the schematic diagram of the functions of the two heterojunctions in NiS_2_/Ni_3_C@C. For Ni_3_C/C heterojunction, its intrinsic properties can construct a space‐charge separation region and promote electron flow from carbon fiber to Ni_3_C to tune the Fermi energy levels, which accelerates the electron/ion transfer during operations. Another NiS_2_/Ni_3_C heterojunction optimizes the active sites and improves the catalytic activity.

**Figure 2:**
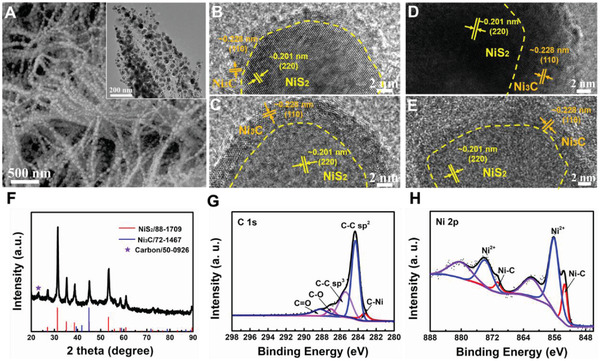
Structural characterizations of NiS_2_/Ni_3_C@C. A) SEM, TEM and B–D) high‐resolution transmission electron microscopy (HRTEM) images of NiS_2_/Ni_3_C@C. F) XRD pattern of NiS_2_/Ni_3_C@C. G–H) X‐ray photoelectron spectroscopy (XPS) spectra of C 1s and Ni 2p.

The structural characterizations are carried out in **Figure** **2**. As observed, the as‐prepared double‐heterojunctional structure shows the peapod‐like morphology with ≈20 nm active nanoparticles embedded along with the carbon fiber, well inherited by the precursor and intermediate products (Figure 2A). The peapod‐like morphology, with active nanoparticles encapsulated inside the carbon fibers, can effectively disperse active materials and protect them from aggregation and peeling off, which can do beneficial for long‐term stability. The corresponding EDS mapping (Figure S5, Supporting Information) presents that Ni, S, and C elements are uniformly distributed in peapod‐like morphology with the atom ratio of ≈2:3:5, suggesting the molar ratio of NiS_2_ and Ni_3_C is ≈9:1 based on the ratio of Ni and S (Figure S6, Supporting Information). By the enlarged HRTEM image in four different regions (Figure 2B–E), we can find the distinct interfaces between NiS_2_ (220) and Ni_3_C (110), confirming the hetero‐structure through in situ chemical reactions. To investigate the formation of Ni_3_C, TEM images of the intermediate products (peapod‐like Ni@C) were provided. As shown in Figure S7, Supporting Information, the lattice spacing of each crystal plane is ≈2.04 Å, consistent with the (111) planes of pure Ni. indicating that Ni_3_C layers can be obtained during sulphuration under the second calcination. Therefore, NiS_2_ may be regarded as an initiator and reactant to form NiS_2_/Ni_3_C heterojunction, which can undoubtedly increase active sites and improve the activity. From the HRTEM image, the Ni_3_C is intimated covered around the NiS_2_, in the middle of the NiS_2_ and carbon, confirming the formation of double‐heterojunctional structure, which can accelerate electron transfer and improve the kinetics. Additionally, there are numerous micropores exist at the interface of NiS_2_/Ni_3_C nanoparticles and carbon fiber, further to facilitate ion fast exchange via electrolyte immersion.

XRD and XPS are performed to investigate the crystal structure and chemical constituent. Figure 2F shows the XRD pattern of NiS_2_/Ni_3_C@C, in which two distinct components of NiS_2_ (88–1709) and Ni_3_C (72–1467) can be disclosed in the composites, in accordance with the HRTEM and EDS results. Herein, a small peak at ≈23° can also be captured, which refers to carbon (50–0926). To further distinguish the components in NiS_2_/Ni_3_C@C, XPS is provided. As for C 1s spectrum (Figure 2G), the peak at ≈283.4 eV corresponds to the Ni‐C bonds, in agreement with the previous literature.^[^ ^15^ ^]^ The peaks at ≈284.4 and ≈285.7 eV refer to sp^2^ and sp^3^ hybridized carbon atoms, respectively. The bonding energies at ≈287 and ≈288.2 eV are indexed to the carbon atoms in the other two functional groups of C–O and C═O, respectively, implying the formation of defected carbon from the glucose, matching well with the Raman and XRD patterns.^[^ ^4^, ^16^ ^]^ In Ni 2p spectrum (Figure 2H), the binding energies of 856.1 and 873.8 eV with the satellite peaks at 861.7 and 880.2 eV, are assigned to 2p_3/2_ and 2p_1/2_ of Ni^2+^ in NiS_2_, separately.^[^ ^17^ ^]^ The strong peaks at 853.2 and 870.5 eV are originated from Ni_3_C.^[^ ^18^ ^]^ The S 2p spectrum observes that the peaks at the binding energies of 162.8 and 164.9 eV are indexed to the 2p_3/2_ and 2p_1/2_ of S_2_^2–^. The peak at 169 eV refers to the SO_4_^2–^ originated from the inevitable oxidation at the surface (Figure S8, Supporting Information). The XRD and XPS patterns further confirm the formation of double‐heterojunctional NiS_2_/Ni_3_C@C.

**Figure 3:**
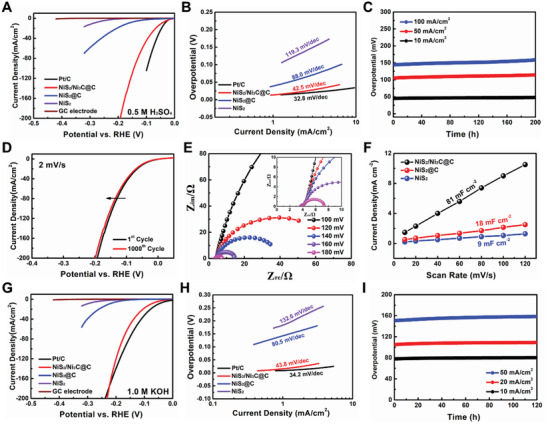
Electrochemical performance of the NiS_2_/Ni_3_C@C toward the hydrogen evolution reaction (HER) in A–F) 0.5 M H_2_SO_4_ and G–I) 1 M KOH. A) Linear sweep voltammetry (LSV) curves of Pt/C, NiS_2_/Ni_3_C@C, NiS_2_@C, and NiS_2_. B) The corresponding Tafel curves are derived from the polarization curves. C) Chronopotentiometry curves of NiS_2_/Ni_3_C@C at various current densities. D) Polarization curves of NiS_2_/Ni_3_C@C before and after 1000 cycles. E) Nyquist plots of NiS_2_/Ni_3_C@ at various overpotentials from 100 to 180 mV. F) Scan rate dependence of the current densities of NiS_2_/Ni_3_C@C, NiS_2_@C, and NiS_2_. G) Linear sweep voltammetry (LSV) curves of Pt/C, NiS_2_/Ni_3_C@C, NiS_2_@C, and NiS_2_. H) The corresponding Tafel curves are derived from the polarization curves. I) Chronopotentiometry curves of NiS_2_/Ni_3_C@C at various current densities.

The HER performance of NiS_2_/Ni_3_C@C was evaluated over a wide pH range. **Figure** **3**A–F shows the electrochemical performance in acidic solution. In the linear sweep voltammetry (LSV) curves (Figure 3A), NiS_2_/Ni_3_C@C delivers an overpotential of 46 mV to reach 10 mA cm^−2^, slightly higher than that of Pt/C catalyst (34 mV), but much lower than NiS_2_@C (164 mV) and NiS_2_ (288 mV). Accordingly, the derived Tafel slope of NiS_2_/Ni_3_C@C is 42.5 mV dec^−1^, almost half of the NiS_2_@C (Figure 3B), indicating that double‐heterojunctional structure can absolutely accelerate HER kinetics. Except for the catalytic activity, long‐term durability is another criterion for catalysts. NiS_2_/Ni_3_C@C shows outstanding stability with an ignorable overpotential increase for 200 h at the constant current density of 10 mA cm^−2^ (Figure 3C). On the contrary, NiS_2_@C reveals an evident increase in overpotential, and NiS_2_ displays a dramatic augment after only 40 h (Figure S9, Supporting Information). When the current density raises to 50 mA cm^−2^ and even 100 mA cm^−2^, there are slightly overpotential increases of 9 and 14 mV, respectively. Except for chronopotentiometry curves, the durability is also confirmed by continuous CV sweeps. Observed in Figure 3D and Figure S10, Supporting Information, the polarization curves almost coincide at various scan rates from 2 mV s^−1^ to 5 and 50 mV s^−1^, suggesting the excellent durability of double‐heterojunctional NiS_2_/Ni_3_C@C, which can be further verified by the SEM image of NiS_2_/Ni_3_C@C after continuous tests (Figure S11, Supporting Information).

Electrochemical impedance spectroscopy is also provided to estimate hydrogen adsorption behavior. The Nyquist plots of NiS_2_/Ni_3_C@C are tested at various overpotentials from 100 to 180 mV, where the charge‐transfer resistance (*R*_ct_) is highly dependent on the overpotential, the higher overpotential, the lower *R*_ct_ (Figure 3E; Figure S12, Supporting Information). Obviously, NiS_2_/Ni_3_C@C exhibits the lowest *R*_ct_ of ≈3.8 Ω at the overpotential of 180 mV than the NiS_2_@C and NiS_2_ of ≈21.5 and ≈78.7 Ω, respectively (Figure S13, Supporting Information), suggesting high faradaic efficiency and rapid electron transfer of NiS_2_/Ni_3_C@C during the process. Furthermore, the Tafel slope value derived from the slope of potential and log *R*_ct_^–1^ is calculated as 43.2 mV dec^−1^ (Figure S14, Supporting Information), accordant with the value obtained from LSV in Figure 3A. Furthermore, electrochemical active surface area (ECSA) is performed to evaluate HER catalytic sites. Figure 3F presents the electrochemical double‐layer capacitance (*C*_dl_) obtained according to the linear slope of the scan rates and the corresponding current densities in CV curves (Figure S15, Supporting Information). As observed, NiS_2_/Ni_3_C@C reveals a higher *C*_dl_ valuer of 71 mF cm^−2^ compared with those of NiS_2_@C (18 mF cm^−2^), NiS_2_ (9 mF cm^−2^). Notably, the calculated ECSA is ≈1775 cm^2^, almost four and eight times as high as that of NiS_2_@C and NiS_2_, supporting the exposed larger active area and active sites for hydrogen adsorption. Remarkably, the eminent performance of NiS_2_/Ni_3_C@C during a wide temperature range is also disclosed. As shown in Figure S16, Supporting Information, low overpotential of 37 and 80 mV can be achieved at 50 and 0 °C, separately, with high stability for 48 h, which indicates the endurance of a wide temperature range for NiS_2_/Ni_3_C@C.

Besides in acidic solution, NiS_2_/Ni_3_C@C also exhibits superior performance in alkaline solution. From LSV curves (Figure 3G), the overpotential of NiS_2_/Ni_3_C@C (*η*_10_ = 78 mV) is much smaller than NiS_2_@C (*η*_10_ = 226 mV) and NiS_2_ (*η*_10_ = 305 mV). Although the *η*_10_ of NiS_2_/Ni_3_C@C is slightly higher than Pt/C (51 mV), the overpotential is smaller than that of Pt/C at the high current density beyond 200 mA cm^−2^, suggesting that NiS_2_/Ni_3_C@C enables high current density with low overpotential, an indicator to estimate the HER catalysts for practical use. Accordingly, the Tafel slope value of NiS_2_/Ni_3_C@C is as low as 43.8 mV dec^−1^. In contrast, NiS_2_@C and NiS_2_ show a higher Tafel slope of 80.5 and 132.6 mV dec^−1^, respectively, implying the desirable and favorable HER kinetics of NiS2/Ni3C@C for H_2_O dissociation and H_2_ generation (Figure 3H). Furthermore, NiS_2_/Ni_3_C@C demonstrates excellent HER performance among recently reported heterogeneous catalysts (Figure S17, Supporting Information) and is even superior to most of the present heterostructure‐based HER electrocatalysts (Table S1, Supporting Information).^[^ ^19^ ^]^ Except for the HER activity, extraordinary stability can also be achieved for NiS_2_/Ni_3_C@C. Noteworthily, NiS_2_/Ni_3_C@C displays a negligible overpotential increase for 120 h at 10 mA cm^−2^. Even under a higher current density of 20 and 50 mA cm^−2^, there are only slightly increases in overpotential for long‐term reactions (Figure 3I). In sharp contrast, a distinct overpotential increase can be observed for NiS_2_@C and NiS_2_ (Figure S18, Supporting Information). Continuous CV sweeps are further carried out to verify the durability of NiS_2_/Ni_3_C@C. As observed, the polarization curves of NiS_2_/Ni_3_C@C almost overlap before and after 1000 cycles at the scan rate of 2 mV s^−1^ (−138 h) (Figure S19, Supporting Information).

The electrochemical tests in neutral solution are further performed to highlight the superiorities of NiS_2_/Ni_3_C@C. In LSV curves, NiS_2_/Ni_3_C@C presents a low *η*_10_ of 91 mV, only a little higher than that of commercial Pt/C catalyst (73 mV), but much lower than that of NiS_2_@C (*η*_10_ = 182 mV) and NiS_2_ (*η*_10_ = 316 mV) (Figure S20A, Supporting Information). In Figure S20B, Supporting Information, NiS_2_/Ni_3_C@C demonstrates the lowest Tafel slope of 58 mV dec^−1^ among the other three electrodes, even outperforming the commercial Pt/C electrode (63.8 mV dec^−1^), significantly implying the rapid reaction kinetics in neutral solution. The efficient catalytic performance in acidic, neutral, and alkaline solutions suggests that NiS_2_/Ni_3_C@C with high active surface area, fast charge/mass transfer, and stable structure can accelerate H_2_O/H adsorption and H_2_O dissociation.

**Figure 4:**
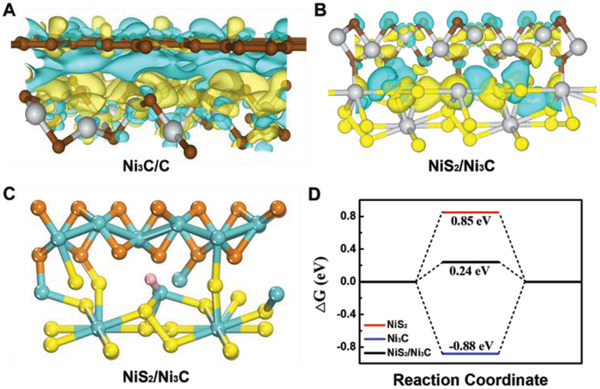
DFT calculations of charge transfer difference and Δ*G*_H*_ of NiS_2_/Ni_3_C@C. Calculated charge transfer difference for A) Ni_3_C/C and B) NiS_2_/Ni_3_C heterojunctions at top views, where the yellow and blue areas refer to the charge accumulation and depletion, respectively. C) The theoretical models used in Δ*G*_H*_. The blue, yellow, brown, and pink balls indicate Ni, S, C, and H atoms, respectively. D) The calculated Gibbs free‐energy diagram of hydrogen evolution reaction (HER) at different active sites.

Double‐heterojunctional electrocatalysts are different from singly‐heterojunctional catalysts, as they can combine the superiorities of two heterojunctions and simultaneously achieve spontaneously fast electron transfer and sufficient high‐active sites for hydrogen absorption/desorption. To confirm and highlight the superiorities, the first‐principle DFT calculations are provided. **Figure** **4**A and Figure S21, Supporting Information show the atomic models of Ni_3_C/C with charge density difference diagrams at the side and top views. As shown, the electron density is significantly rearranged at the interface and the electrons are transferred from carbon fiber to Ni_3_C, indicating the Ni_3_C/C heterojunction trigger spontaneous electron diffusion. Furthermore, from the electronic DOS of Ni_3_C and carbon, we can detect that Ni_3_C presents metal‐like properties while defected carbon exhibits semiconductor‐like properties (Figure S22, Supporting Information). It is obvious that Ni_3_C/C heterojunction can create a Schottky barrier,^[^ ^6a^ ^]^ indicating the spontaneous electron flow from the outer carbon fibers to Ni_3_C and accessible to catalytic active sites, beneficial for fast electron/proton diffusion.

For NiS_2_/Ni_3_C heterojunction (Figure 4B, S23, Supporting Information), there is an evident increase in interfacial charge density, implying the strong interaction between NiS_2_ and Ni_3_C. The concentrated localized charge density further confirms that the catalytic active center moves to the interface, and the electron accumulation can do a favor for electron‐deficient hydrogen adsorption, leading to improved HER performance.^[^ ^10a^ ^]^ To verify the high catalytic activity, Gibbs's free energy of hydrogen adsorption (Δ*G*_H*_) is provided. It is well accepted that the HER activity is highly related to Δ*G*_H*_. Commonly, an ideal value of Δ*G*_H*_ should be zero both for easy adsorption and release. Figure 4C and Figure S24, Supporting Information, present the hydrogen adsorption configurations of pure NiS_2_, Ni_3_C, and NiS_2_/Ni_3_C heterostructure based on the experimental results (HRTEM image). As observed, hydrogen was absorbed on the interface between (220) planes of NiS_2_ and (113) planes of Ni_3_C. In the DFT results (Figure 4D), NiS_2_/Ni_3_C heterostructure exhibits a more optimized Δ*G*_H_ of (0.24 eV) when compared with that of NiS_2_ (0.85 eV) and Ni_3_C (−0.88 eV), suggesting a desirable hydrogen adsorption–desorption behavior for NiS_2_/Ni_3_C@C. The DFT calculations of charge transfer difference and Δ*G*_H*_ sufficiently confirm that Ni_3_C/C and NiS_2_/Ni_3_C heterojunctions in NiS_2_/Ni_3_C@C can accelerate electron fast and spontaneous transfer, change the active sites and enhance the activity. Therefore, NiS_2_/Ni_3_C@C can undoubtedly exhibit high HER performance.

## 3 Conclusion

In summary, we develop a nanoscale double‐heterojunctional NiS_2_/Ni_3_C@C as a proof‐of‐concept to optimate the charge/mass transfer and active sites of electrocatalysis. The experimental characterizations confirm the formation of double heterointerfaces among NiS_2_, Ni_3_C, and Carbon. Owing to the strong interaction between the intimately contacted three components, the porous double‐heterojunctional nanostructure is successfully achieved. The DFT calculations elucidate that the Ni_3_C/C heterojunction accelerates electron/mass flow accessible to catalytic active sites, and NiS_2_/Ni_3_C heterojunction enriches the active sites and enhances the catalytic activity. The nanoscale double‐heterojunctional NiS_2_/Ni_3_C@C is fabricated as a durable and effective HER electrocatalyst and delivers low overpotentials of 46, 91, and 78 mV with small Tafel slopes of 42.5, 58, 43.8 mV dec^−1^ at 10 mA cm^−2^ in acidic, neutral and alkaline conditions, respectively, which outperforms most of the recently reported primary heterostructural electrocatalysts. The porous double‐heterojunctional nanostructure offers a new multistructural model for high‐HER‐performance catalysts and may provide a structural paradigm for other catalytic reactions, such as ORR/OER and NRR, especially under acidic conditions.

## Conflict of Interest

The authors declare no conflict of interest.

## Supporting information

Supporting Information

## Data Availability

Research data are not shared.
