# Supplementary material for: Nanoscale Double‐Heterojunctional Electrocatalyst for Hydrogen Evolution
Source: Adv Sci (Weinh). 2022 Apr 24;9(18):2201339. doi: 10.1002/advs.202201339 (PMC9218783; doi:10.1002/advs.202201339)
Supplement: Supplementary file 1 — Supporting Information [file ADVS-9-2201339-s001.pdf]

ADVANCED SCIENCE
Open Access

Supporting Information

for *Adv. Sci.*, DOI 10.1002/advs.202201339

Nanoscale Double-Heterojunctional Electrocatalyst for Hydrogen Evolution

*Yangyang Feng, Yongxin Guan, Enbo Zhou, Xiang Zhang and Yaobing Wang**

Supporting Information

Nanoscale Double-heterojunctional Electrocatalyst for Hydrogen Evolution

Yangyang Feng[1#], *Yongxin Guan*[2#], *Enbo Zhou*[1], *Xiang Zhang*[1], *Yaobing Wang*[1,3,4,*]

[1] CAS Key Laboratory of Design and Assembly of Functional Nanostructures, and Fujian Provincial Key Laboratory of Nanomaterials, State Key Laboratory of Structural Chemistry, Fujian Institute of Research on the Structure of Matter, Chinese Academy of Sciences, Fuzhou 350002, Fujian, P. R. China.

[2]Chongqing Industry Polytechnic College, Chongqing, 401120, P. R. China

[3]Fujian Science and Technology Innovation Laboratory for Optoelectronic Information of China, Fuzhou 350108, Fujian, P. R. China

[4]University of Chinese Academy of Sciences, Beijing 100049, P. R. China

#These authors contributed equally to this work

Experimental procedures

Materials:

Sodium hydroxide (NaOH, Aldrich, 99.9%), Nickel sulfate ($NiSO_4$, Aldrich, 99.9%), Glucose (Cica-Reagent, Kanto Chemical), Thiourea (Aldrich, 99.0%).

Preparation of α-$Ni(OH)_2$ nanowires

At first, Sodium hydroxide (9.8 mmol) and Nickel sulfate (9.8 mmol) were uniformly mixed and added in D.I. water (35 mL). After stirring for 20 min, we transferred the mixture into the autoclave (50 mL) for hydrothermal reaction (160 ^oC, 3 h). After naturally cooling down, the green sample was washed and dried.

Preparation of Ni@C and NiS_2/Ni_3C@C nanocomposites

α-$Ni(OH)_2$ (100 mg) and glucose (0.5 mM) were mixed in deionized water (40 mL). The solution after uniform mixing was transferred into autoclave for carbon coating

(180 ^{o}C, 8 h). Then, cleaning and washing the as-obtained intermediate products by D.I. water and ethanol, and drying overnight at 60 ^{o}C. Afterwards, the sample was put into tube furnace under calcination in Ar to form peapod-like Ni@C under 700 ^{o}C for 3 h. Then, the peapod-like Ni@C was annealed again with thiourea under 450 ^{o}C for 120 min in Ar to change Ni into NiS_2.

Characterization

The morphology characterizations were investigated by scanning electron microscope (SEM, FEI, JSM-7800F) under the voltage of 5 kV, energy dispersive spectrometer analyzer (EDS), and transmission electron microscope (TEM, Tecnai, 300 kV). The phase and structure characterizations are tested by X-ray diffractometer (XRD, Bruker ECO D8 power) under Cu Ka with the 2 theta of 10-90°, X-ray photo electron spectrometer (XPS, ESCALAB 250Xi, Thermo scientific, 225 W, 15 mA, 15 kV Al Kα), Raman microscope (Renishaw Invia, laser: 532 nm) and BET specific-surface-area pore-size analyzer (Quantachrome Autosorb-6B) and.

Electrochemical Tests:

Electrochemical properties were performed on CHI760 workstation by three-electrode setup, where saturated calomel electrode (SCE), carbon rod, and the as-prepared sample on glassy carbon (3 mm) were used as reference, counter and working electrodes, separately under acidic (0.5 M H_2SO_4), alkaline (1.0 M KOH) and neutral (1M PBS) conditions. The working electrode was obtained by 4 mg NiS_2/Ni_3C@C, NiS_2@C and NiS_2, 0.1 mL Nafion solution and 0.3 mL ethanol solvent. There was 4 μL solution (~0.42 mg/cm^2 mass loading) on the electrode.

Electrochemical LSV tests were conducted from -0.5 to 0 V. The CV profiles were measured from 0 to 0.2 V. AC impedance measurements were performed from 10^5-0.1 Hz at various voltages. In all measurements, the data was according to the Nernst equation: $E_{(RHE)} = E_{(SCE)} + 0.0592\ pH + 0.241$.

ECSA in this work was calculated by electrochemical double layer capacitence (C_{dl}) values via the following equation:

$$\mathrm{ECSA} = \frac{C_{dl}}{C_s}$$

C_s refers to the specific capacitance which is calculated through smooth planar surface. The specific capacitance on the smooth surface is accepted to be during 20 to 60 μF/cm^2. In this manuscript, we assumed 40 μF/cm^2.

Theoretical calculations

Calculations in this work were performed using the CASTEP code by Vienna ab Initio Simulation package (VASP) [1]. The interaction between core and valence electrons is presented by standard PAW [2] potentials. The correlation functional and electron-electron exchange were described via Perdew-Burke-Ernzerhorf generalized gradient approximation (PBE-GGA) [3]. The electron wave function was in plane-wave with the cutoff energy of 450 eV in all computations with the force threshold of 0.01 eV/ Å.

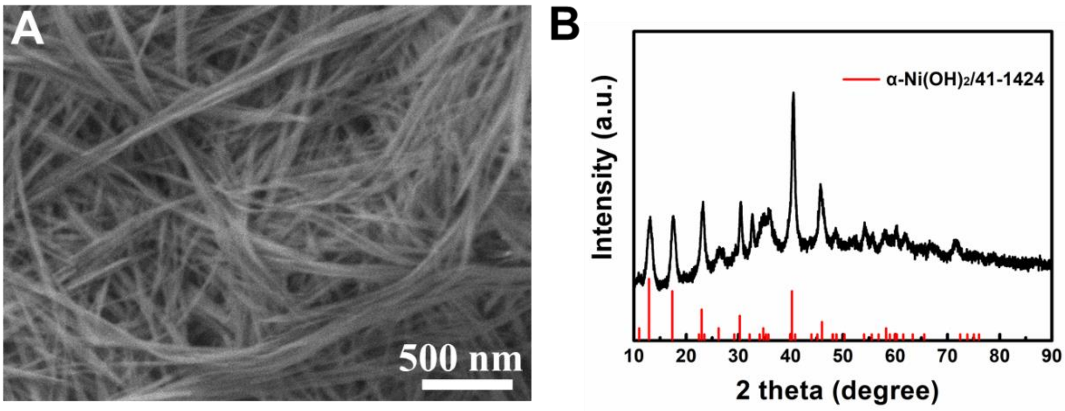

Figure S1. (A) XRD patterns and (B) SEM image of α-$Ni(OH)_2$, in which that 1D α-$Ni(OH)_2$ nanowires with an average thickness of 20-40 nm can be largely and evenly synthesized through a common hydrothermal method.

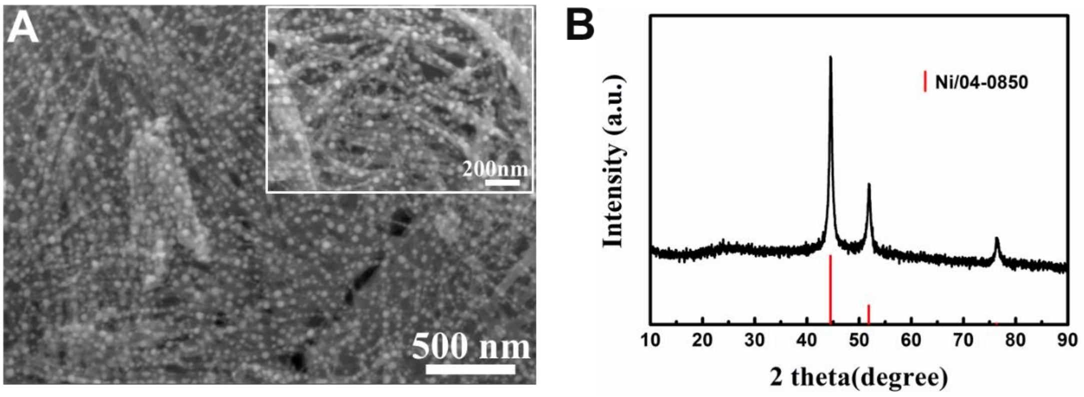

Figure S2. (A) XRD patterns and (B) SEM image of peapod-like Ni@C.

Through in-situ carbon coating and calcination, α-$Ni(OH)_2$ can be self-assembly transferred into Ni nanoparticles along with the 1D wire-like structure, while glucose changes into graphitized carbon fiber. From the SEM images (Figure S2A), we can detect that numerous active nanoparticles are tightly confined in carbon fibers, with a certain space between each particle, forming numerous Ni/C heterojunctions.

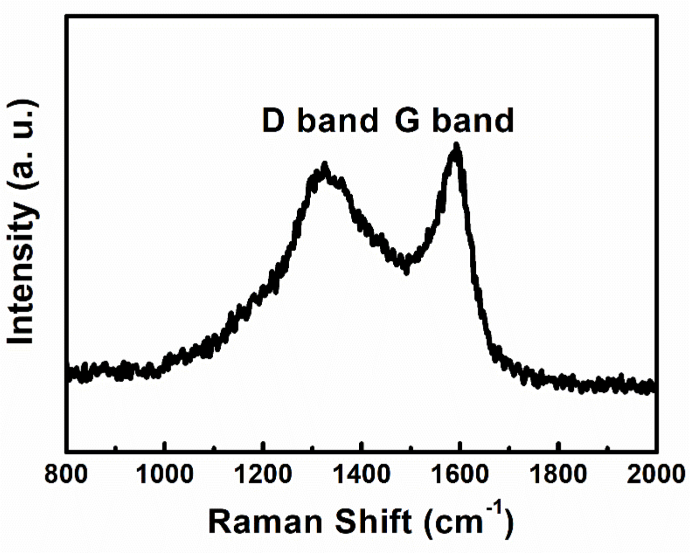

Figure S3. Raman spectrum of $NiS_2/Ni_3C@C$.

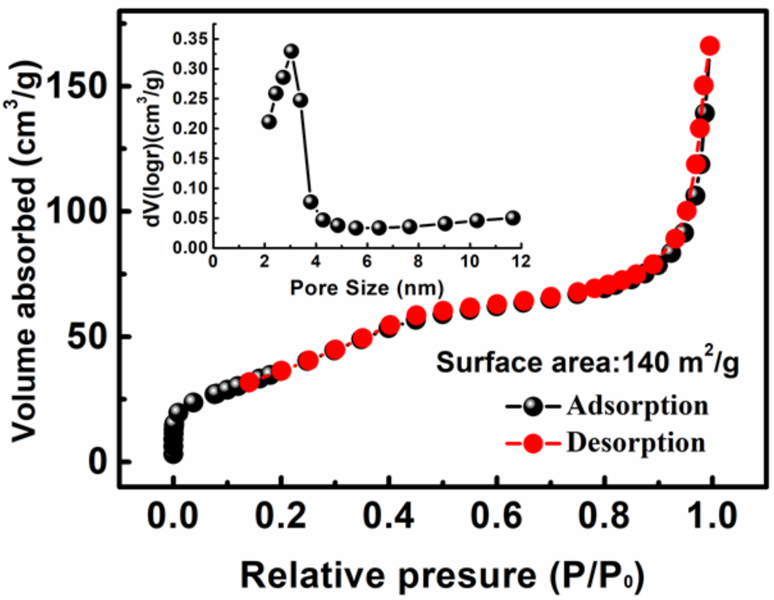

Figure S4. BET profile of $NiS_2/Ni_3C@C$ to reveal the specific surface area and pore size distribution (inset) derived from the desorption branch.

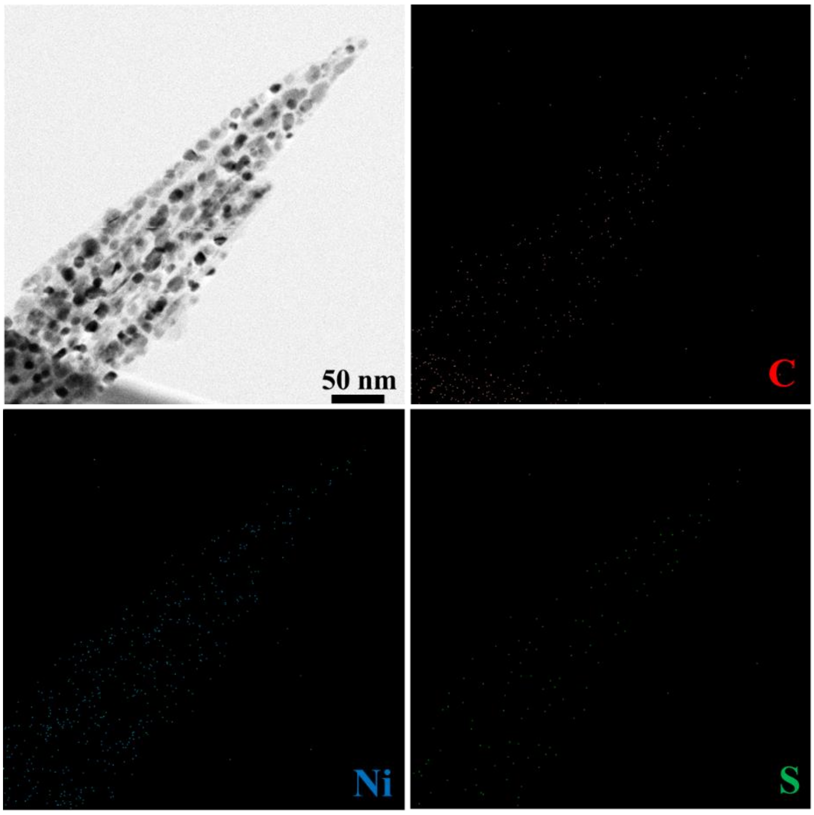

Figure S5. The corresponding EDS mapping of $NiS_2/Ni_3C@C$.

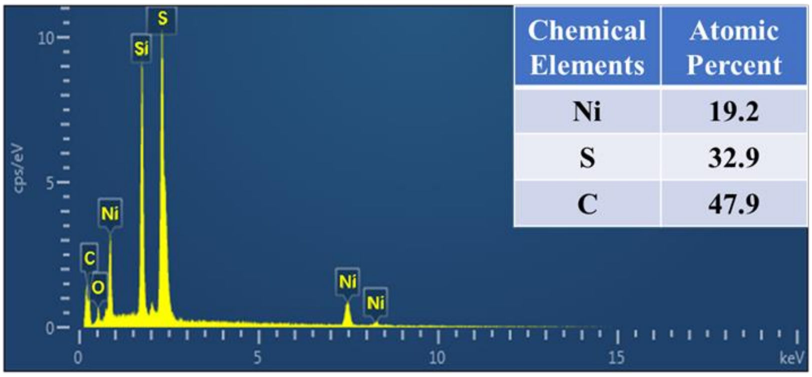

Figure S6. The corresponding EDS analysis of $NiS_2/Ni_3C@C$.

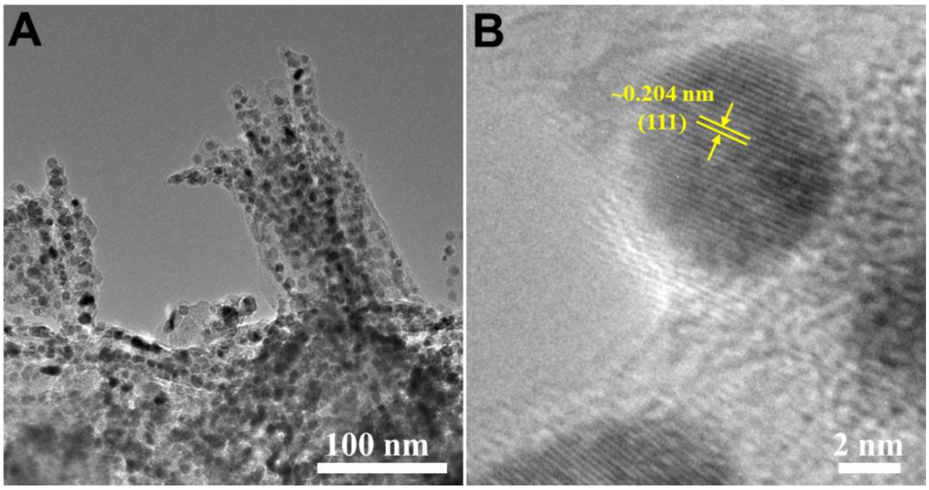

Figure S7. (A) TEM and (B) HRTEM images of peapod-like Ni@C.

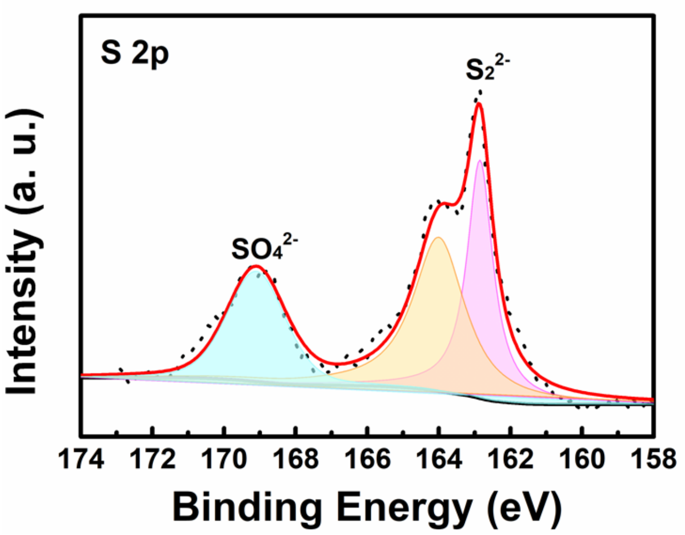

Figure S8. XPS spectrum of S 2p in $NiS_2/Ni_3C@C$.

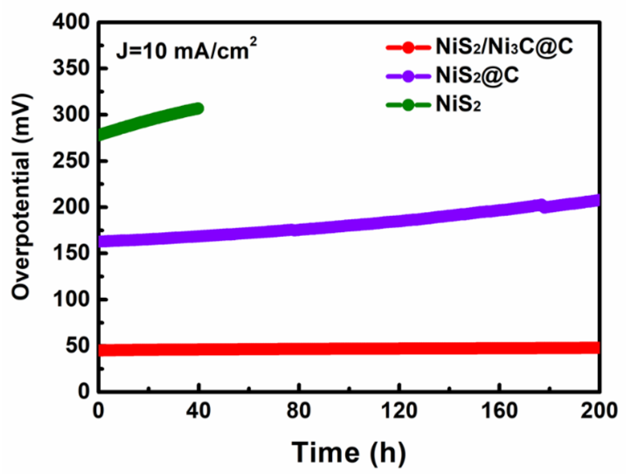

Figure S9. Chronopotentiometry curves of $NiS_2/Ni_3C@C$, $NiS_2@C$ and NiS_2 at the current density of 10 mA/cm^2 in 0.5 M H_2SO_4.

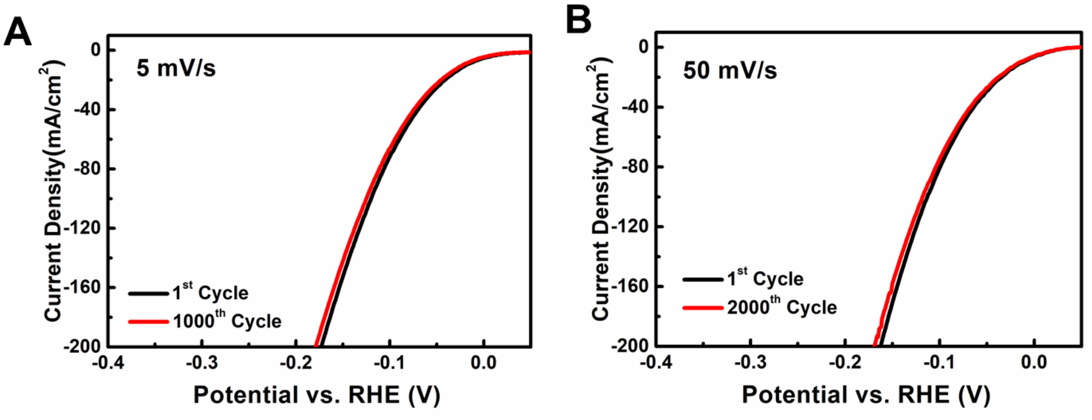

Figure S10. Electrochemical durability of $NiS_2/Ni_3C@C$ at the scan rates of (A) 5 and (B) 50 mV/s.

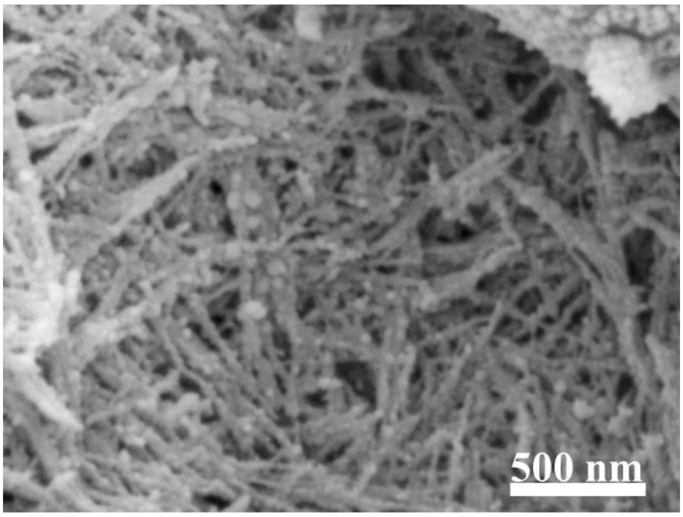

Figure S11. SEM image of $NiS_2/Ni_3C@C$ after running for 200 h.

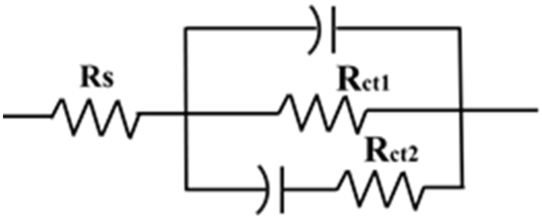

Figure S12. The fitted equivalent circuit of $NiS_2/Ni_3C@C$.

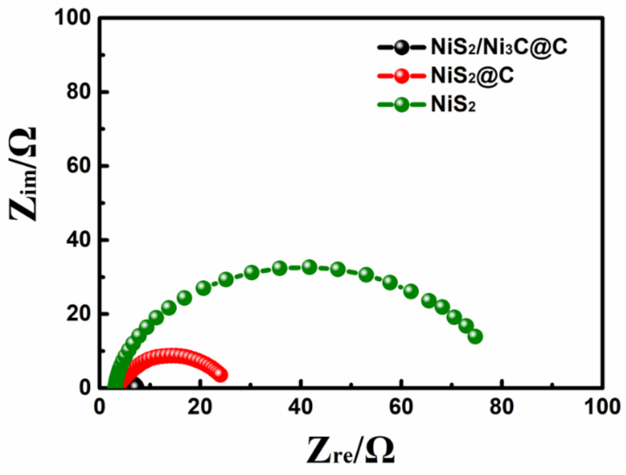

Figure S13. Nyquist plots of $NiS_2/Ni_3C@C$, $NiS_2@C$ and NiS_2 tested at the same overpotential of 180 mV.

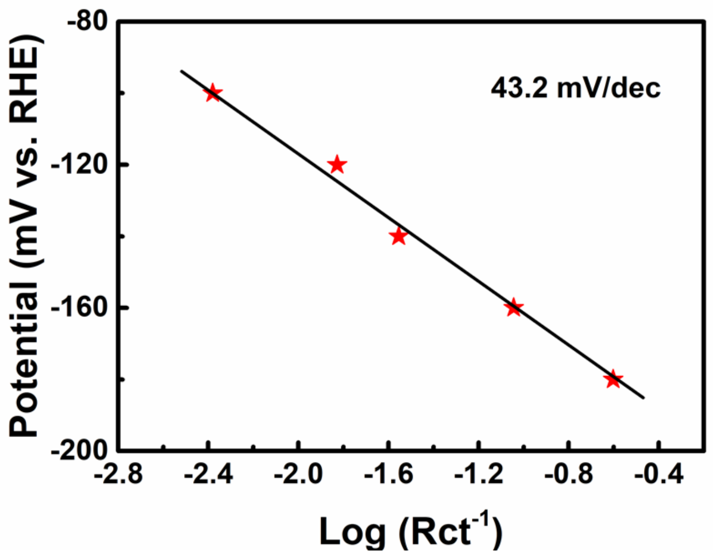

Figure S14. Linear plot of potential vs. log (R_{ct}^{-1}) for $NiS_2/Ni_3C@C$.

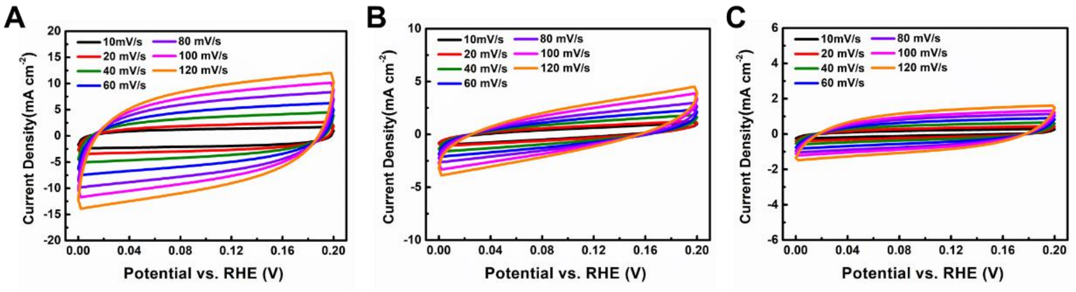

Figure S15. CV of (A) $NiS_2/Ni_3C@C$, (B) $NiS_2@C$ and (C) NiS_2 tested at various scan rates from 10 to 120 mV/s in the potential range of 0-0.2 V.

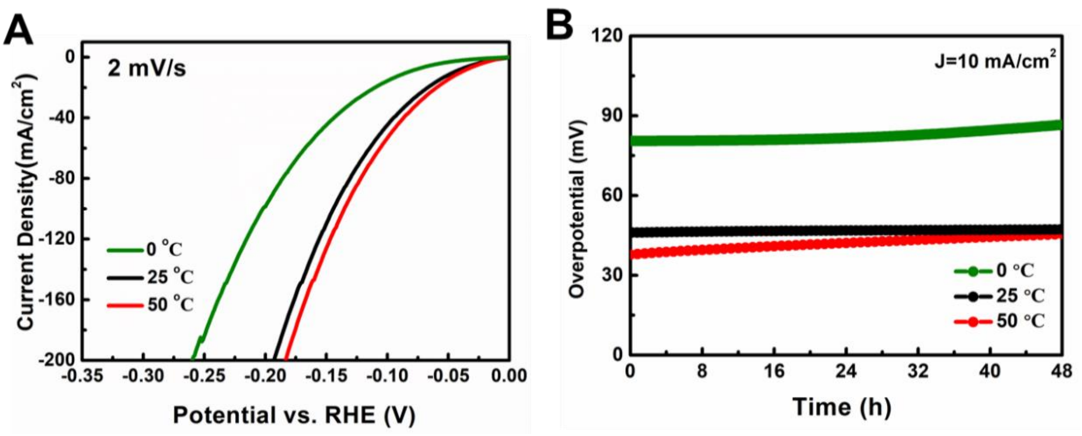

Figure S16. Temperature effect of $NiS_2/Ni_3C@C$ tested in 0.5 M H_2SO_4. (A) Polarization curves tested at 0, 25 and 50 °C at a scan rate of 2 mV/s. (B) Durability test at 0, 25 and 50 °C at a constant current density of 10 mA/cm^2.

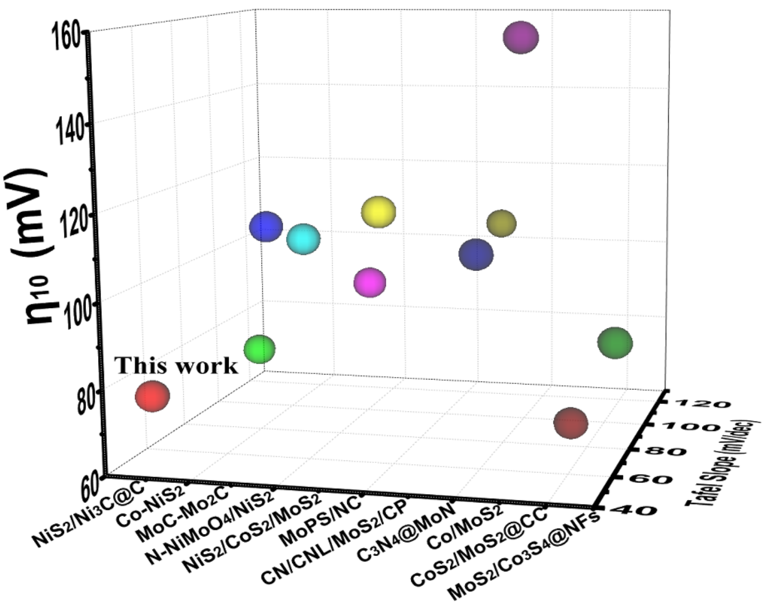

Figure S17. The comparison of overpotentials at 10 mA cm^{-2} and Tafel slopes between $NiS_2/Ni_3C@C$ and recently reported heterogeneous HER electrocatalysts in 1 M KOH.

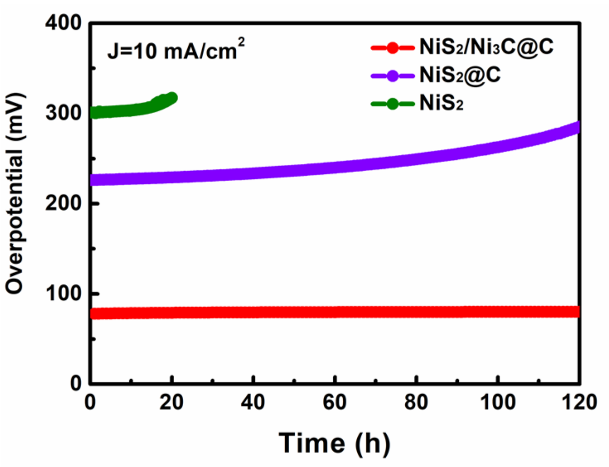

Figure S18. Chronopotentiometry curves of $NiS_2/Ni_3C@C$, $NiS_2@C$ and NiS_2 at the current density of 10 mA/cm^2 in 1 M KOH.

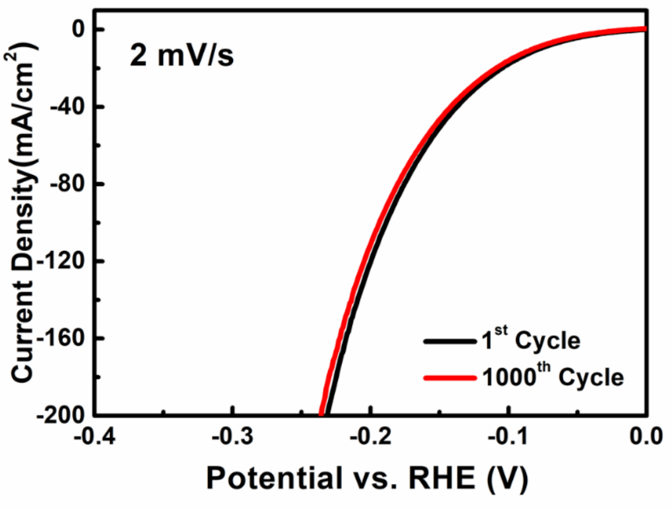

Figure S19. Electrochemical durability of $NiS_2/Ni_3C@C$ at the scan rate of 2 mV/s in alkaline solution.

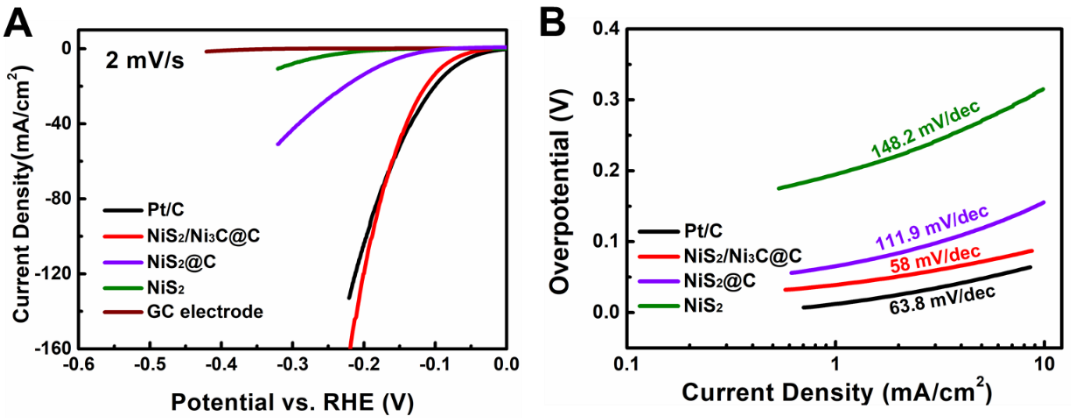

Figure S20. (A) Linear sweep voltammetry (LSV) curves and (B) Tafel slopes of Pt/C, $NiS_2/Ni_3C@C$, $NiS_2@C$, NiS_2 in 1M PBS.

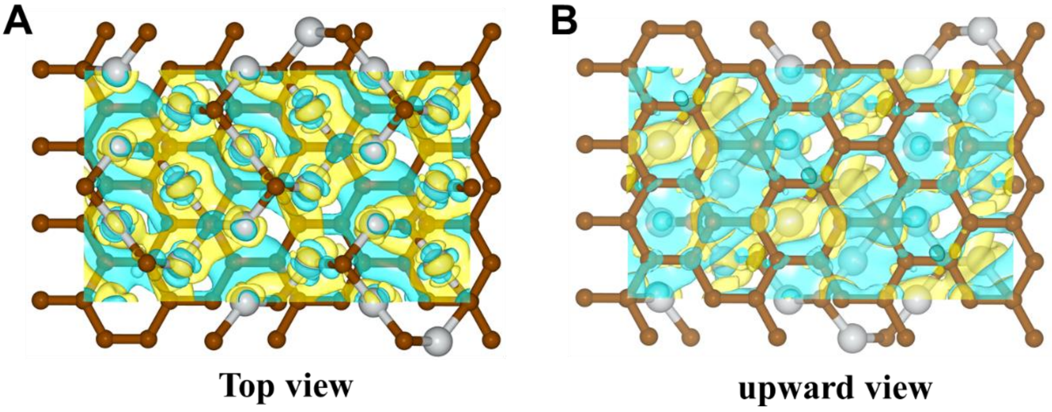

Figure S21. Atomic models of Ni_3C/C with charge density difference diagrams at (A) top and (B) upward views.

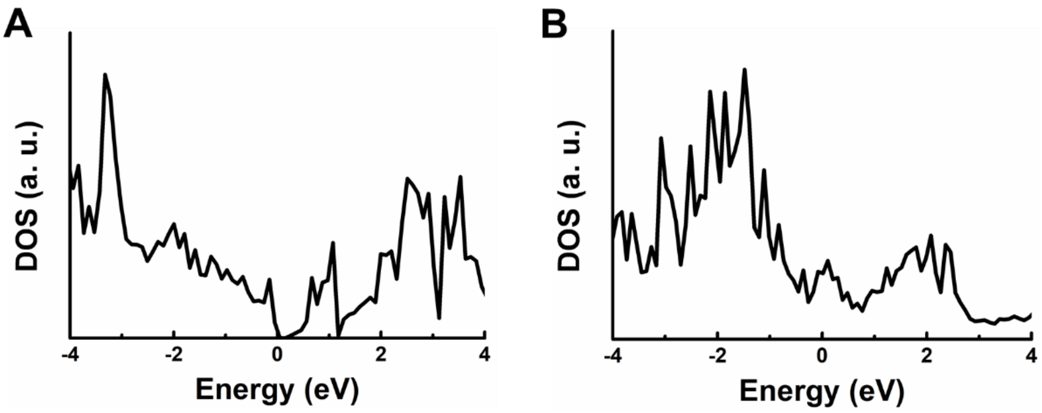

Figure S22. Density of states (DOS) of (A) defected carbon and (B) Ni_3C.

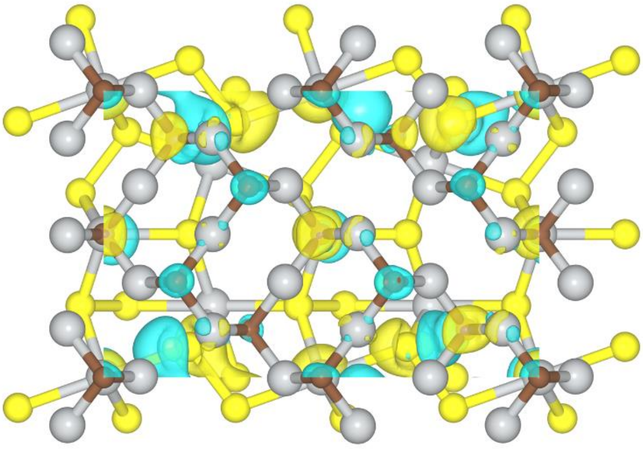

Figure S23. Atomic models of NiS_2/Ni_3C with charge density difference diagrams at top views.

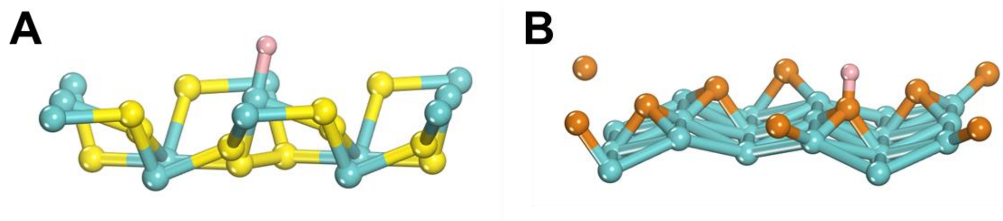

Figure S24. The hydrogen adsorption configurations of pure (A) NiS_2, (B) Ni_3C, where the blue, yellow, brown and pink balls indicate Ni, S, C and H atoms, respectively.

Table S1. Summary of some recently reported heterogeneous HER catalysts in alkaline electrolytes.

Catalyst	η_{10} (mV vs. RHE)	Tafel slope (mV/dec)	Ref.
**NiS_2/Ni_3C@C**	78	43.8	This work
**Co-NiS_2 NSs[4]**	80	80	Angew. Chem. Int. Ed. 2019, 58, 18676
**MoC-Mo_2C[5]**	114	62	Nat. Commun. 2021, 12, 6776
**$NiS_2/CoS_2/MoS_2$[6]**	112	59	Sci. Bull. 2020, 65, 359
**N-$NiMoO_4/NiS_2$[7]**	99	74.2	Adv. Funct. Mater. 2019, 29, 1805298
**MoP@NC[8]**	149	61.7	Appl. Catal. B: Environ., 2020, 263, 118358
**MoPS/NC[9]**	120	52	Appl. Cataly. B: Environ. 2019, 245, 656
**CN/CNL/MoS_2/CP[10]**	106	117	Chem. Eng. J. 2021, 412, 128556
**C_3N_4@MoN[11]**	110	57.8	Nano Energy 2018, 53, 690.
**Co/MoS_2[12]**	158	58	Nano Energy 2017, 39, 409.
**CoS_2/MoS_2@CC[13]**	71	62.8	ChemSusChem, 2021, 14, 669

**Edge-MoS$_2$/Co$_3$S$_4$@NFs**[14]	90.3	61.69	Mater. Today Energy 2020, 18, 100513

Supplemental references

[1] G. Kresse, D. Joubert, *Phys.l Rev. B* **1999**, 59, 1758-1775.

[2] P. E. Blöchl, *Phys. Rev. B* **1994**, 50, 17953-17979.

[3] J. P. Perdew, K. Burke, M. Ernzerhof, *Phys. Rev. Lett.* **1996**, 77, 3865-3868.

[4] C.-T. Dinh, A. Jain, F. P. G. de Arquer, P. De Luna, J. Li, N. Wang, X. Zheng, J. Cai, B. Z. Gregory, O. Voznyy, B. Zhang, M. Liu, D. Sinton, E. J. Crumlin, E. H. Sargent, *Nat. Energy* **2019**, 4, 107-114.

[5] W. Liu, X. Wang, F. Wang, K. Du, Z. Zhang, Y. Guo, H. Yin, D. Wang, *Nat. Commun.* **2021**, 12, 6776.

[6] Y. Zhang, M. Shi, C. Wang, Y. Zhu, N. Li, X. Pu, A. Yu, J. Zhai, *Sci. Bull.* **2020**, 65, 359-366.

[7] L. An, J. Feng, Y. Zhang, R. Wang, H. Liu, G.-C. Wang, F. Cheng, P. Xi, *Adv. Funct. Mater.* **2019**, 29, 1805298.

[8] C. Pi, C. Huang, Y. Yang, H. Song, X. Zhang, Y. Zheng, B. Gao, J. Fu, P. K. Chu, K. Huo, *Appl. Catal. B: Environ.* **2020**, 263, 118358.

[9] Y. Huang, X. Song, J. Deng, C. Zha, W. Huang, Y. Wu, Y. Li, *Appl. Catal. B: Environ.* **2019**, 245, 656-661.

[10] J. Dong, X. Zhang, J. Huang, J. Hu, Z. Chen, Y. Lai, *Chem. Eng. J.* **2021**, 412, 128556.

[11] H. Jin, X. Liu, Y. Jiao, A. Vasileff, Y. Zheng, S.-Z. Qiao, *Nano Energy* **2018**, 53, 690-697.

[12] X. Hai, W. Zhou, S. Wang, H. Pang, K. Chang, F. Ichihara, J. Ye, *Nano Energy* **2017**, 39, 409-417.

[13] G. Zhou, X. Wu, M. Zhao, H. Pang, L. Xu, J. Yang, Y. Tang, *ChemSusChem* **2021**, 14, 699-708.

[14] O. Peng, R. Shi, J. Wang, X. Zhang, J. Miao, L. Zhang, Y. Fu, P. Madhusudan, K. Liu, A. Amini, C. Cheng, *Mater. Today Energy* **2020**, 18, 100513.
